# Venetoclax combined with chidamide and decitabine successfully induced remission in relapsed/refractory classical Hodgkin lymphoma: a case report and literature review

**DOI:** 10.3389/fimmu.2026.1757010

**Published:** 2026-05-15

**Authors:** Xuelian Jin, Yun Tang, Yu Wu, Jie Ji

**Affiliations:** Department of Hematology, West China Hospital, Sichuan University, Chengdu, Sichuan, China

**Keywords:** Bcl-2 inhibitor (Venetoclax), CHL, demethylation (decitabine), epigenetic regulation, histone deacetylase inhibitor (chidamide), relapsed/refractory, targeted therapy

## Abstract

Most patients with classical Hodgkin lymphoma (cHL) can achieve long-term survival with frontline combination chemotherapy and radiation therapy, but approximately 10%-30% of patients will develop relapsed/refractory (R/R) disease. High-dose chemotherapy followed by autologous stem cell transplantation (ASCT) combined with brentuximab vedotin (BV) maintenance therapy improves patient prognosis. However, the management of relapse after ASCT remains a challenge. This report presents a case of a patient with relapsed cHL after ASCT who was successfully treated with targeted therapy combined with two epigenetic modulators. A 26-year-old male patient was diagnosed with stage IIA cHL and received five-line treatment, including ABVD, GemOx-D, anti-PD-1/PD-L1 monoclonal antibodies, anti-CD47/PD-L1 bispecific antibodies, and BV combined with ICE, ultimately achieving first complete remission (CR). The patient subsequently underwent peripheral blood ASCT and BV maintenance therapy. However, 16 months post transplantation, disease relapse was detected on follow-up PET/CT scan. The patient subsequently received 6 cycles of the Ven–Chi–Dec regimen (venetoclax combined with chidamide and decitabine), achieving complete metabolic remission (CMR) with no significant adverse effects. This case report suggests that the Ven–Chi–Dec regimen may be a potential treatment for patients with R/R cHL after ASCT, although further studies are needed to validate its efficacy.

## 1 Introduction

Patients with newly diagnosed classical Hodgkin lymphoma (cHL) generally have a favorable prognosis following first-line treatment, with approximately 75% of patients achieving 3–5 years of progression-free survival (PFS) (1). However, 10%-30% of patients may develop relapsed/refractory (R/R) disease (2, 3). High-dose salvage chemotherapy followed by autologous stem cell transplantation (ASCT) is the standard treatment for these patients. However, approximately 40%-50% of patients still experience progressive disease (PD) following transplantation (4, 5). Studies have shown that maintenance therapy with brentuximab vedotin (BV) after ASCT significantly improves outcomes for this high-risk group (6); however, effective treatments for relapse after ASCT are limited. This report presents a patient with primary refractory cHL who achieved his first complete remission (CR) after five lines of treatment and later relapsed following peripheral blood ASCT and BV maintenance. He ultimately achieved complete metabolic remission (CMR) through the Ven–Chi–Dec regimen (venetoclax, chidamide and decitabine), which combines a targeted drug with dual epigenetic regulations.

## 2 Case description

**Figure 1:**
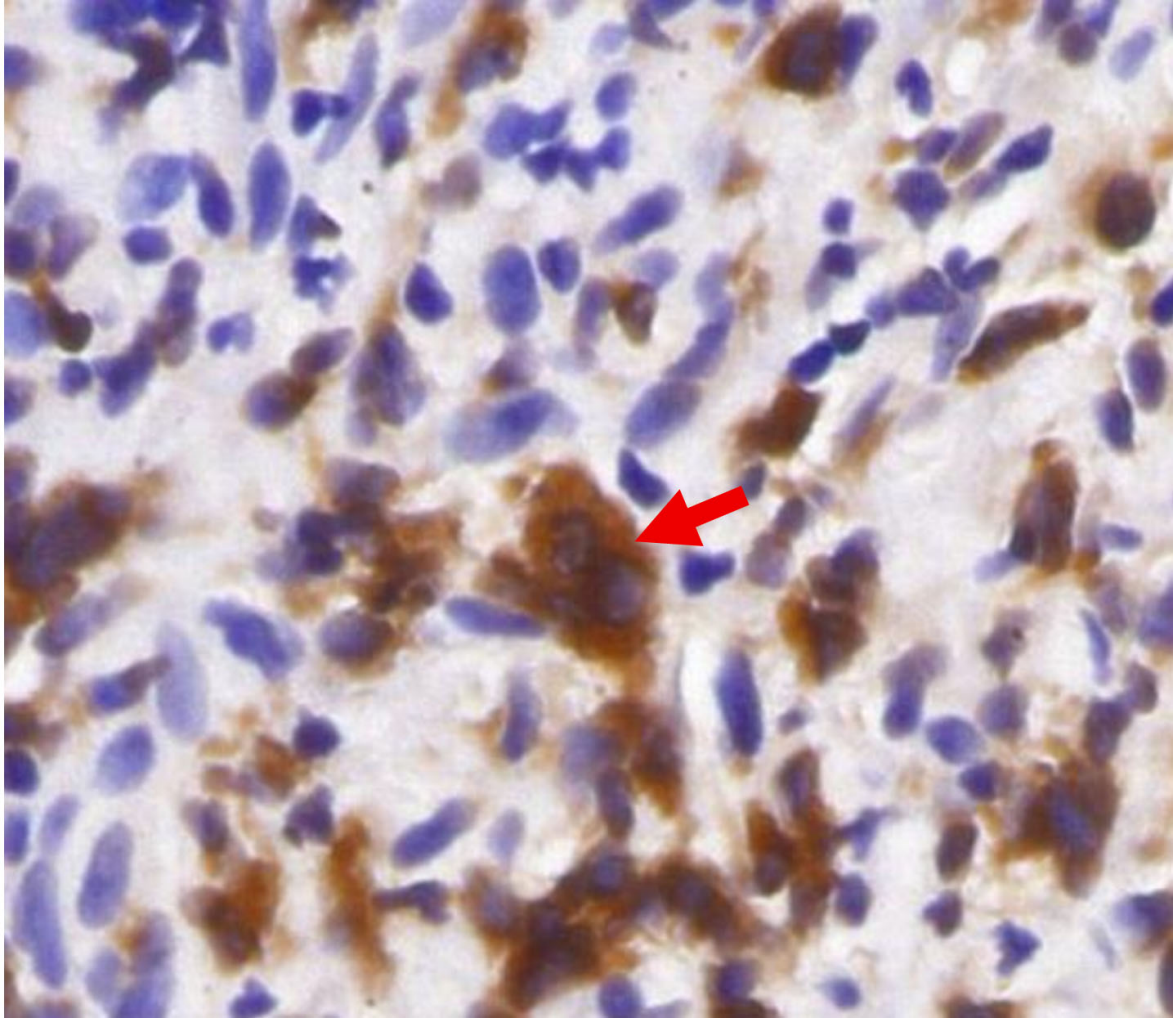
BCL−2 immunohistochemical staining of the lymph node specimen. The red arrow highlights BCL−2–positive HRS cell.

A 26-year-old male patient presented in March 2018 with multiple enlarged lymph nodes above the diaphragm, including the bilateral supraclavicular fossae; axillary, hilar, mediastinal, and bilateral cardiophrenic angles; and internal mammary lymph nodes. Histopathological examination following excisional biopsy of the lymph nodes in the right supraclavicular fossa revealed a diagnosis of cHL (stage IIA). Immunohistochemical analysis revealed that the tumor cells were CD45^-^CD20^-^CD3^-^CD30^+^CD15(+, in small amounts) BCL-2^+^EBV^-^PAX-5^dim+^MUM1^+^ALK^-^EMA^-^GB^-^ and EBER1/2 negative *in situ* hybridization. BCL−2 immunohistochemical staining of the lymph node specimen is shown in **Figure 1**. He received six cycles of ABVD regimen chemotherapy (doxorubicin, bleomycin, vinblastine, and dacarbazine). However, follow-up PET/CT imaging revealed PD. In September 2018, after one cycle of GemOx-D (gemcitabine, oxaliplatin, and dexamethasone) as second-line therapy, contrast-enhanced CT scans continued to indicate PD. In November 2018, the patient was subsequently enrolled in an open-label, multicenter phase II clinical trial (NCT03580564) evaluating the safety and efficacy of KL-A167 injection, an anti-PD-L1 monoclonal antibody, for R/R cHL. Despite completing six cycles of treatment, the PET/CT scan still revealed PD. In February 2019, the patient initiated nivolumab therapy and completed a total of 44 treatment cycles. Follow-up PET/CT in January 2022 again indicated PD. In March 2022, the patient was enrolled in a phase I clinical trial (NCT04795128) to assess the safety, tolerability, and preliminary antitumor activity of IBI322, an anti-CD47/PD-L1 bispecific antibody, in patients with cHL who had demonstrated resistance to anti-PD-1/PD-L1 monoclonal antibodies. After 24 weeks of treatment, a PET/CT scan continued to demonstrate PD.

**Figure 2:**
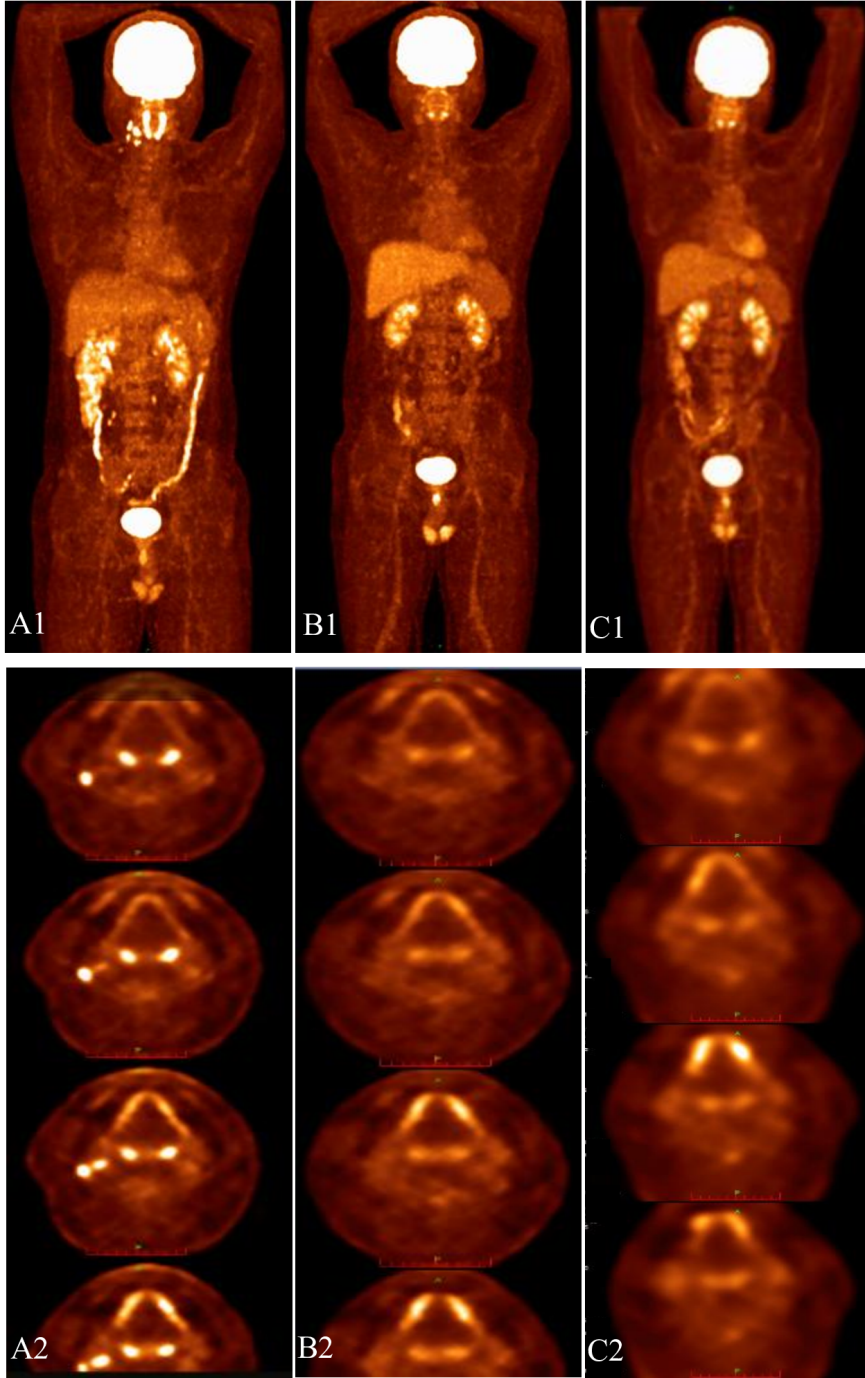
Comparison of PET/CT before and after treatment with the regimen. PET/CT revealed active 18F-FDG metabolism in the right cervical lymph nodes before Ven–Chi–Dec treatment **(A1, A2)**. PET/CT scan revealed CMR after Ven–Chi–Dec treatment **(B1, B2)**. Follow−up PET/CT (September 2025) demonstrating sustained CMR **(C1, C2)**.

**Figure 3:**
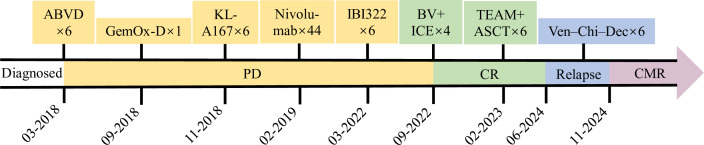
Summary of the antitumor treatments received by the patient since diagnosis in March 2018.

In September 2022, the patient underwent four cycles of BV combined with ICE (ifosfamide, carboplatin, and etoposide), and a subsequent PET/CT scan revealed CMR (Deauville score 3). In February 2023, he underwent autologous peripheral blood stem cell transplantation with a conditioning regimen of etoposide, cytarabine, and melphalan (TEAM). Posttransplantation, the patient received 11 cycles of BV maintenance therapy. A PET/CT scan performed in June 2024 revealed an increase in both the size and number of lymph nodes in the right cervical region, accompanied by elevated glucose metabolism (maximum SUV: 12.92), suggesting disease relapse (**Figures 2A1, A2**). The patient subsequently received six cycles of Ven–Chi–Dec therapy (venetoclax 100 mg orally on days 1–28, chidamide 20 mg orally biweekly for 4 weeks, and decitabine 10 mg subcutaneously on days 1–5). During the Ven–Chi–Dec treatment period, the patient remained asymptomatic without fever, cough, fatigue, bleeding, or vomiting. During treatment, complete blood count monitoring revealed a minimum hemoglobin concentration of 104 g/L (CTCAE grade 1), a white blood cell count of 2.2 × 10^9^/L (CTCAE grade 2), an absolute neutrophil count of 0.9 × 10^9^/L (CTCAE grade 2), and a platelet count of 70 × 10^9^/L (CTCAE grade 2). No transfusion support was needed. A follow-up PET/CT scan on November 20, 2024, revealed a CMR (Deauville score of 1) (**Figures 2B1, B2**). The patient subsequently received consolidation therapy with the Ven–Chi–Dec regimen. A follow-up PET/CT scan performed on September 9, 2025, continued to show CMR (Deauville score 1) (**Figures 2C1, C2**). The chidamide dose in the regimen was then reduced to 10 mg for maintenance therapy. **Figure 3** provides a summary of the patient’s entire antitumor treatment course since diagnosis, and the patient remained disease-free thereafter.

## 3 Discussion

To our knowledge, this is the first case of combining a BCL-2 inhibitor with epigenetic modulators in the treatment of relapsed cHL following multiple lines of chemotherapy. The patient, who was diagnosed with primary refractory cHL, achieved CR after receiving five-line therapy but subsequently experienced disease relapse after ASCT and BV maintenance therapy. Ultimately, CMR was achieved through combination therapy involving venetoclax, chidamide, and decitabine.

Although ASCT following salvage chemotherapy can result in long-term disease-free survival in approximately 50% to 60% of patients with R/R cHL (4, 7), managing patients who relapse post-ASCT remains a significant clinical challenge due to limited therapeutic options. For R/R cHL patients who previously received ASCT, BV, and anti-PD-1 therapy, low-intensity chemotherapy regimens based on bendamustine and gemcitabine may be considered. However, these regimens generally yield only transient responses, as the disease often develops resistance over time. Additionally, oral mTOR inhibitors such as everolimus and immunomodulatory agents such as lenalidomide may be explored. However, their CR rates are generally less than 10% (8).

cHL is a B-cell-derived malignancy characterized by the presence of Hodgkin and Reed–Sternberg (HRS) cells. Over 60% of cHL patients express BCL-2, which contributes to inhibiting apoptosis (9, 10). Gamboa-Cedeno et al. demonstrated that cell lines derived from patients with primary refractory or progressive cHL exhibited notable sensitivity to the BCL-2 inhibitor venetoclax, suggesting its potential as a viable therapeutic strategy for this patient population (10). Nevertheless, HRS cells frequently exhibit loss of canonical B-cell markers (e.g., CD20 and CD79a), primarily due to extensive abnormal epigenetic alterations, including promoter hypermethylation, epigenetic silencing, and aberrant expression of polycomb group proteins (11). Histone deacetylase inhibitors (HDACis) can enhance the immune response against Hodgkin lymphoma or exert antitumor proliferation effects through various epigenetic regulatory mechanisms, such as upregulating P21 and OX40 ligands, downregulating STAT6 and CCL17, and activating the caspase pathway. Several clinical studies have demonstrated that HDACis exhibit significant antitumor activity in patients with relapsed Hodgkin lymphoma, including patients who relapse after ASCT (12, 13).

Chidamide, an HDACi, has demonstrated synergistic activity when combined with venetoclax and can overcome drug resistance and improve response rates. In R/R diffuse large B-cell lymphoma (DLBCL) and R/R acute myeloid leukemia (AML), studies have indicated that chidamide enhances the antitumor effects of venetoclax by modulating the expression of MCL-1, BCL-2, and BIM, thereby producing a pronounced synergistic effect (14, 15).

Decitabine, a DNA demethylating agent, also has synergistic antilymphoma effects when used in combination with venetoclax and/or chidamide. Although single-agent decitabine shows limited efficacy in advanced malignancies, low-dose administration has been associated with enhanced T-cell function and increased chemosensitivity (16). Furthermore, similar to HDACis, low-dose decitabine, can also upregulate the expression of caspase-8, thereby increasing tumor sensitivity to apoptosis (17). Clinical trials have shown that in R/R cHL, the combination of decitabine (10 mg/day on days 1-5) with chidamide and camrelizumab achieves an objective response rate of up to 94% (18). In DLBCL, decitabine potentiates the effects of venetoclax by inhibiting the PI3K-AKT signaling pathway, altering the mitochondrial membrane composition, inducing DNA damage responses, and enhancing BAX and BAK activity, leading to synergistic suppression of lymphoma cell proliferation (19). Our team previously reported successful treatment of a patient with extramedullary relapse of R/R B-ALL who presented with lymphadenopathy and subcutaneous masses via a combination of azacitidine, venetoclax, and chidamide, resulting in the resolution of lymphadenopathy and subcutaneous masses (20).

On the basis of the aforementioned preclinical and clinical evidence, we hypothesized that the combination of venetoclax, chidamide, and decitabine would also demonstrate synergistic antitumor activity in cHL. The clinical outcome was grade 3/4 promising, as the patient achieved CMR without experiencing any hematologic or systemic adverse events during treatment. This case provides preliminary evidence supporting the potential efficacy of the Ven–Chi–Dec regimen in treating R/R cHL. Nonetheless, certain limitations should be acknowledged, including that a confirmatory lymph node biopsy to assess BCL−2 status at relapse was not performed and that findings from a single−case report remain inherently limited. Further studies are warranted to validate these findings.

## Data Availability

The raw data supporting the conclusions of this article will be made available by the authors, without undue reservation.
